# Retroperitoneal Hemorrhage Due to Spontaneous Renal Rupture as the First Presentation of Antiphospholipid Syndrome: A Case Report

**DOI:** 10.7759/cureus.36839

**Published:** 2023-03-29

**Authors:** Charalampos Mavridis, Eleni Lagoudaki, Georgios Georgiadis, Athanasios Bouchalakis, Charalampos Mamoulakis

**Affiliations:** 1 Urology, University General Hospital of Heraklion, University of Crete, Medical School, Heraklion, GRC; 2 Pathology and Laboratory Medicine, University General Hospital of Heraklion, University of Crete, Medical School, Heraklion, GRC

**Keywords:** spontaneous retroperitoneal hemorrhage, emergency department, retroperitoneal hematoma, antiphospholipid syndrome, spontaneous renal rupture

## Abstract

Spontaneous renal rupture (SRR) with retroperitoneal hemorrhage is an extremely rare medical emergency and is rather challenging for the surgical team. Management of SRR often requires surgical intervention and nephrectomy as it is life-threatening. Antiphospholipid syndrome (APLS) is an autoimmune disease that affects several organs, including kidneys, causing significant abnormalities. Current data suggest that APLS can result in renal artery stenosis, renal vein thrombosis, arterial hypertension, thrombotic microangiopathy, and antiphospholipid syndrome nephropathy where there is renal involvement. Here, we report the case of a 49-year-old man who presented to the Emergency Department with sudden-onset abdominal pain in the context of retroperitoneal bleeding due to SRR. The patient developed hemodynamic instability and underwent a total nephrectomy. The surgical specimen revealed APLS-related lesions. Serological tests confirmed the diagnosis of APLS, which was managed with acenocoumarol and hydroxychloroquine. Since then, he has not experienced any thromboembolic or hemorrhagic episodes. This article aims to present for the first time a case of SRR as the first presentation of APLS as well as to analyze the possible associated mechanisms.

## Introduction

Spontaneous retroperitoneal hemorrhage-hematoma (SRHH) and spontaneous renal rupture (SRR) are rare medical entities mandating urgent treatment as they can be fatal [1,2]. Nevertheless, their common clinical manifestation is abdominal pain which is not a pathognomonic finding [1,3]. Often, these patients present to the emergency department in a state of hemorrhagic shock [1,3]. The origin of retroperitoneal bleeding and the identification of the underlying cause of SRR constitute serious diagnostic challenges [1,3]. Non-traumatic retroperitoneal hemorrhage originates predominantly from the psoas muscle, followed by the kidney [1]. The most common pathology of the kidney leading to spontaneous bleeding is the presence of a tumor (benign or malignant) [2,3]. Antiphospholipid syndrome (APLS) is an autoimmune disease that causes thrombosis in the involved organs [4]. APLS can affect the kidney causing renal artery stenosis, arterial hypertension, antiphospholipid syndrome nephropathy (APLSN), and thromboembolic lesions [4]. The diagnosis of APLS requires the measurement of anticardiolipin antibodies (a-CL), β2 glycoprotein I antibodies (a-β2GPI), and lupus anticoagulant (LA) in the serum of a patient who has had a thromboembolic event [5]. This report aims to present a unique case of a 49-year-old man with SRHH-SRR related to primary, new-onset APLS.

This case report was previously presented as a meeting abstract at the 25th Panhellenic Urological Congress on October 6, 2022.

## Case presentation

**Figure 1:**
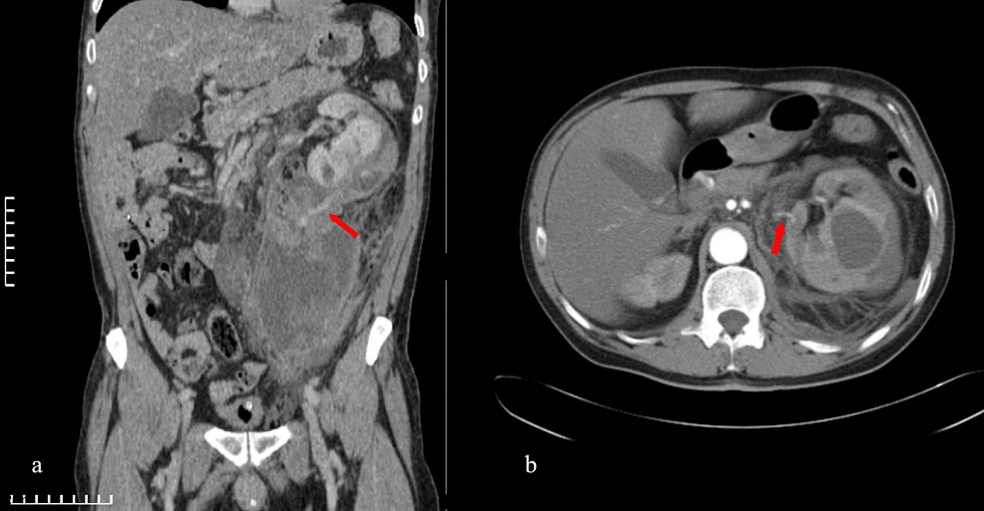
Renal rupture with active retroperitoneal bleeding and hematoma (a, b). Grade IV renal rupture on the left side according to the latest American Association for the Surgery of Trauma (AAST) renal injury grading scale [6]. The red arrows show active bleeding. (a) CT, arterial phase, coronal view, abdomen. (b) CT, arterial phase, horizontal view.

A 49-year-old male patient presented to the emergency department with acute, sudden-onset abdominal pain without other comorbid symptoms. The pain was widespread across the abdomen and extended to the scrotum. It was of intense severity as the patient spoke with difficulty and seemed unable to process the questions asked during the clinical examination well. He did not report any recent injury, and his medical history revealed no pathologies. During the clinical examination, he developed mild tachycardia (103 beats/minute), maintaining normal blood pressure; mean arterial pressure was 74 mmHg, while his abdominal wall was in contraction. Immediate bedside ultrasound was performed, revealing a large retroperitoneal mass (hematoma) on the left. His initial hemoglobin (Hb) level was low (11.1 g/dL). He had an abnormal serum creatinine (Crea) level (1.8 mg/dL) and 10 red blood cells per high-power field (HPF) in his urine sample. Furthermore, the urine spot test revealed a 2+ grade of proteinuria based on turbidity. The international normalized ratio (INR) and platelets were normal. The patient received prompt fluid resuscitation as well as blood product transfusion with two packed red blood cell (PRBC) units and one fresh frozen plasma (FFP) unit. Following our resuscitation, the patient was stable enough to undergo a contrast-enhanced computed tomography (CT) scan. The CT scan revealed an SRHH with a grade IV SRR on the left side, according to the latest American Association for the Surgery of Trauma (AAST) renal injury grading scale (Figures 1a, 1b) [6].

**Figure 2:**
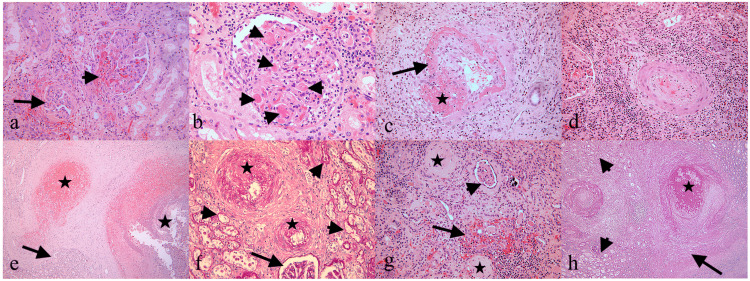
Antiphospholipid syndrome nephropathy (a-h). a. A glomerulus with eosinophilic fibrinoid thrombi occluding the vascular pole (arrowhead) alongside an ischemic glomerulus with tuft retraction (arrow). b. Closer view at a glomerulus showing multiple thrombi in the capillary lumens (arrowheads), thickened capillary walls, and swollen endothelial cells. c. An arcuate artery with zones of fibrinoid deposits (arrow) superimposed on preexisting myointimal hyperplasia and partial luminal occlusion by eosinophilic fibrinoid material (star). d. An arteriole with myofibroblastic hyperplasia of the intima and proliferative changes in the media. e. Arteries with luminal thrombi consisting of red blood cell fragments, leukocytes, and eosinophilic fibrinoid material (stars) bordering areas of interstitial fibrosis and tubular thyroidization, with the latter consisting of atrophic tubules with eosinophilic casts (arrow). f. Periodic acid–Schiff stain highlights the grade of fibrous hyperplasia of the intima (stars) of two arterioles with narrowed lumens, the fibrotic basement membranes of atrophic tubules (arrowheads), as well as the fibrosis of the glomerular tufts (arrow). g. Atrophic renal cortex showing a pair of entirely fibrotic glomeruli (stars), a glomerulus with shrunken ischemic tuft, and enlarged Bowman’s space (arrowhead) besides an area of tubular thyroidization (arrow). h. Interlobular arteries with severe myointimal hypertrophy one of which with luminal thrombus (star) alongside areas of tubular atrophy (arrowheads) and interstitial fibrosis (arrow) decorated with periodic acid–Schiff stain. Hematoxylin and eosin stain (a, d, f, g) ×200, (b, c) ×400, (e, h) ×50 magnification.

The patient became hemodynamically unstable, with a rapidly decreasing Hb level (8 g/dL); hence, he was admitted urgently to the operating theater. An open surgical incision with a retroperitoneal approach was chosen to investigate the bleeding. Intraoperatively, detecting the site of active bleeding was not feasible due to the large perinephric and retroperitoneal hematoma. The most notable finding was the presence of a large blood clot mass in the retroperitoneum. In addition, it was difficult to identify the ureter, and the surgical preparation of the kidney was laborious. Finally, a left-open nephrectomy was performed without any complications, and the patient was stabilized. During his hospitalization, he received nine PRBC and five FFP units. On the seventh postoperative day, he was discharged in good clinical status, with Hb of 11.2 g/dL and Crea of 2 mg/dL. Histopathologic examination of the surgical specimen of the excised kidney revealed lesions compatible with APLSN, consisting of the presence of combined acute and chronic vascular lesions secondary to thrombotic microangiopathy (TMA) affecting the glomeruli, preglomerular arterioles, and small interlobular arteries, as well as their chronic ischemic sequelae in the interstitium and renal tubules. Glomeruli insulted by APLS developed thrombi composed of eosinophilic fibrinoid material along with fragments of red blood cells and leukocytes which occlude the hilum and the capillary lumina. Besides the thrombi, endothelial swelling and foci of non-inflammatory fibrinoid necrosis of the respective walls pose an additional risk for tubular occlusion. Tubules with simple epithelium with nuclear reactive atypia reflecting acute tubular injury were also present. Chronic TMA glomerular lesions consisted of bloodless glomeruli with tuft retraction and segmental and global glomerulosclerosis. Arterioles with acute TMA damage showed endothelial swelling, myointimal proliferation, media thickening, thrombi, and areas of fibrinoid necrosis of their walls without evidence of inflammatory infiltration (vasculitis was absent). On the other hand, chronic TMA lesions consisted of intimal fibrosis, varying luminal narrowing, and recanalized thrombi. The renal cortex exhibited areas of parenchymal atrophy characterized by interstitial fibrosis and zones of atrophic tubules containing eosinophilic casts. Such changes mimicked thyroid parenchyma (tubular thyroidization) (Figures 2a-2h).

The patient underwent thrombophilia screening, revealing high-titer IgG a-CL and a-β2GPI (106.6 U/mL and 101.3 U/mL, respectively) beyond three months, while LA, antinuclear antibodies (ANA), and extractable nuclear antigen antibodies (ENA) were negative. ENA screen detects antibodies against Smith protein (anti-SM), ribonucleoprotein (anti-RNP), histidyl tRNA synthetase (anti-Jo-1), topoisomerase 1 (anti-Scl-70), ribonucleoprotein molecules SS-A (anti-Ro), and SS-B (anti-La). Moreover, our patient did not develop any constitutional or other clinical symptoms associated with systematic erythematosus lupus (SLE). Based on the Sapporo criteria, a diagnosis of primary APLS was made [5]. Since then, the patient has been on treatment with acenocoumarol (AC) and co-administered hydroxychloroquine (HCQ). After six years of regular follow-up, he has not experienced any thromboembolic or bleeding events, and his Crea level has stabilized at 1.5 mg/dL. Moreover, the Hb level was higher than 14 g/dL. Table 1 summarizes the normal reference range and cut-offs for the laboratory tests mentioned in this report.

**Table 1:** Results of blood tests and reference values considered by our laboratory. Hb: hemoglobin; Crea: creatinine; HPF: high-power field; a-CL: anticardiolipin antibodies; a-β2GPI: β2 glycoprotein I antibodies; LA: lupus anticoagulant; anti-dsDNA: double-stranded DNA antibodies; ANA: antinuclear antibodies; ENA: extractable nuclear antigen; ED: emergency department; PO: preoperation; PD: postoperative day; n/a: not applicable *: ENA screen detects antibodies against Smith protein (anti-SM), ribonucleoprotein (anti-RNP), histidyl tRNA synthetase (anti-Jo-1), topoisomerase 1 (anti-Scl-70), ribonucleoprotein molecules SS-A (anti-Ro) and SS-B (anti-La).

Laboratory test	Reference range (units)	ED	PO	PD1	PD7	PD100
Hb	14–18 g/dL (men)	11.1	8	10.1	11.2	14.1
Crea	0.7–1.3 mg/dL (men)	1.8	1.7	2.2	2	1.5
Red blood cells per HPF (urine sample)	<3	10	n/a	n/a	1	<1
Protein grade (urine sample, based on turbidity)	Negative	2+	n/a	n/a	1+	traces
IgG a-CL	<20 U/mL	n/a	n/a	n/a	n/a	106.6
IgG a-β2GPI	<20 U/mL	n/a	n/a	n/a	n/a	101.3
LA (LA screen/LA confirm)	<1.2	n/a	n/a	n/a	n/a	1.12
anti-dsDNA	<5 U/mL	n/a	n/a	n/a	n/a	2.4
ANA	<1:80	n/a	n/a	n/a	n/a	<1:80
ENA screen*	<20 U/mL	n/a	n/a	n/a	n/a	3.3

## Discussion

Abdominal pain is probably the most common reason for presenting to the emergency department [7]. The differential diagnosis of non-traumatic abdominal pain in men includes a variety of pathologies involving the gastrointestinal system, urinary tract, large vessels, and musculoskeletal system [8]. Nevertheless, in cases with coexisting signs of hypovolemic shock, SRHH should be considered [9]. Ultrasonography is valuable in investigating abdominal pain, especially in examining the retroperitoneal space [9]. Ultrasound provides immediate information that helps physicians establish an accurate diagnosis and manage patients more accurately. The common disadvantage is that it is highly dependent on the examiner’s experience [10]. In our case, the assessment was performed by a urologist certified in ultrasonography. Therefore, SRHH was immediately identified without clarifying the origin of the bleeding. However, there was a high suspicion that it originated from the left kidney as the normal renal window was absent. Because the patient remained hemodynamically stable, we decided that contrast-enhanced CT should be performed so that treatment options could be targeted. Because the bleeding originated from the left kidney, we proceeded with the surgical approach when the patient developed hemodynamic instability. One of the most common causes of SRR is the presence of a malignancy [2,3]. Thus, surgical treatment should be preferred compared to selective embolization, even in the case of a hemodynamically stable patient. The histopathological findings of our case were those of vascular nephropathy characterized by the combination of acute and chronic TMA vascular lesions affecting glomerular capillaries and arteries as well as areas of interstitial fibrosis and tubular atrophy without evidence of cancer. These findings are consistent with APLSN [4]. Similar features can be present in a patient with SLE [4]. Unfortunately, immunofluorescence microscopy was not performed for the detection of IgG, IgM, IgA, C3, C1q, and fibrin deposits as the nephrectomy specimen was placed into formalin. Despite this, our patient had no suspicious clinical findings such as oral or nasal ulcers, arthritis, serositis, malar or discoid rash, photosensitivity, and any neurologic disorder. Regarding the obtained hematological tests three months after the nephrectomy, there was no leukopenia, thrombocytopenia, or anemia. Moreover, anti-dsDNA, ANA, LA, and ENA antibodies were negative, while IgG a-CL and a-β2GPI remained positive in the serum. Even in rare cases of true ANA-negative SLE, anti-Ro/SSA autoantibodies are positive [11]. The clinical suspicion for SLE was extremely low in our case as clinical presentations were absent, and anti-dsDNA, ANA, and ENA antibodies were negative. Considering the aforementioned, the diagnosis of primary APLS was established.

Turrent-Carriles et al., in their recent review, studied the possible renal involvement in APLS [4]. Theoretically, in APLS, infractions or thrombosis can occur in any renal vessel [4]. Furthermore, the disease is associated with the development of hypertension and chronic kidney failure [4]. Nevertheless, the essential query remains: which mechanisms can trigger renal bleeding in APLS? The existing literature reveals that patients with lupus nephritis and TMA or APLS or positive a-CL and/or a-β2GPI and/or LA carry a higher risk of bleeding after renal biopsy [4]. This observation was concluded from a retrospective patient data analysis without clarifying the mechanism [12]. Our patient’s history did not reveal any renal injury, a fact that can support an increased risk of bleeding in such a scenario. A possible explanation for the SRR would be the establishment of thrombocytopenia, as it can occur in up to 50% of patients with APLS [5]. As we noted, our patient did not develop thrombocytopenia during hospitalization. Another potential explanation for SRR would be the occurrence of a hypertensive crisis. Of significant note, 93% of patients with APLSN develop hypertension; thus, a hemorrhagic event can be triggered [4]. Bleeding can also be induced due to ischemic morphological alterations caused by TMA and other vascular lesions with the presence of fragile tissues [2,3]. Furthermore, prolonged ischemia can lead to endothelial dysfunction and disruption of endothelial cell boundaries, causing a bleeding event [13]. Finally, a sudden increase in renal venous pressure due to post-occlusive reactive hyperemia can result in renal rupture [3,14]. As underlined by the histopathological report, the presented vascular lesions in the kidney of our patient could have triggered some of the aforementioned ischemia-related mechanisms causing SRR. However, the exact mechanism is unclear; therefore, it is necessary to investigate the association of APLS with bleeding on a clinical and molecular basis.

The patient’s follow-up has been uneventful, as he has not experienced another thromboembolic or bleeding event. His renal function has also not been compromised further. Regarding his standard treatment, the target INR between 2 and 3 was achieved by taking AC 2 mg every Monday and Thursday and 1 mg on the remaining days of the week. The co-adjuvant use of HCQ may have positively contributed to the outcome of the case so far [15]. The patient was informed of all possible complications related to HCQ and consented to its administration. He was treated with 200 mg of HCQ daily in the first year. Thereafter, he received 200 mg twice a week. The use of HCQ may offer a protective role in preventing the recurrence of thrombotic events in patients with APLS without associated SLE; however, in the absence of large-scale prospective or randomized studies, this approach could not be clearly justified [15]. Because the case presented a severe thrombotic-hemorrhagic complication due to APLS, we considered the potential benefit greater than the cost. Moreover, the dose of HCQ the patient receives is relatively low, and so far, he has not experienced any HCQ-related adverse event. Annual cardiac check-ups and periodic blood tests remain normal. To our knowledge, this is the first case of SRHH/SRR due to APLS.

## Conclusions

APLS affects the kidney through many different known and unknown mechanisms. SRR can be a potential complication of APLS under specific conditions, such as hypertension, thrombocytopenia, and ischemia, but the exact triggering mechanisms remain unclear. A high titer of a-CL and/or a-β2GPI antibodies in our patient probably exacerbated or caused renal bleeding. In SRR cases, and in the absence of underlying pathologies such as a tumor, it may be necessary to check for autoimmune diseases and thrombophilia. Further research is required to identify the molecular mechanisms that can contribute to a hemorrhage episode in the context of APLS.
